# Semantic fMRI neurofeedback of emotions: from basic principles to clinical applications

**DOI:** 10.1098/rstb.2023.0084

**Published:** 2024-10-21

**Authors:** Rainer Goebel, Michael Lührs, Assunta Ciarlo, Fabrizio Esposito, David E. Linden

**Affiliations:** ^1^ Department of Cognitive Neuroscience, Faculty of Psychology and Neuroscience, Maastricht University, Oxfordlaan 55, Maastricht 6229 EV, The Netherlands; ^2^ Research Department, Brain Innovation BV, Oxfordlaan 55, Maastricht 6229 EV, The Netherlands; ^3^ Department of Medicine, Surgery and Dentistry, ‘Scuola Medica Salernitana’, University of Salerno, S. Allende 43, Baronissi (SA) 84081, Italy; ^4^ Department of Advanced Medical and Surgical Sciences, School of Medicine, University of Campania ‘Luigi Vanvitelli’, Piazza Luigi Miraglia 2, Naples 80123, Italy; ^5^ Department of Psychiatry & Neuropsychology, School for Mental Health and Neuroscience, Faculty of Health, Medicine and Life Science, Maastricht University, Universiteitssingel 40, Maastricht 6229 ER, The Netherlands

**Keywords:** fMRI, neurofeedback, representational similarity analysis (RSA), self-regulation, emotion

## Abstract

During fMRI neurofeedback participants learn to self-regulate activity in relevant brain areas and networks based on ongoing feedback extracted from measured responses in those regions. This closed-loop approach has been successfully applied to reduce symptoms in mood disorders such as depression by showing participants a thermometer-like display indicating the strength of activity in emotion-related brain areas. The hitherto employed conventional neurofeedback is, however, ‘blind’ with respect to emotional content, i.e. patients instructed to engage in a specific positive emotion could drive the neurofeedback signal by engaging in a different (positive or negative) emotion. In this future perspective, we present a new form of neurofeedback that displays semantic information of emotions to the participant. Semantic information is extracted online using real-time representational similarity analysis of emotion-specific activity patterns. The extracted semantic information can be provided to participants in a two-dimensional semantic map depicting the current mental state as a point reflecting its distance to pre-measured emotional mental states (e.g. ‘happy’, ‘content’, ‘sad’, ‘angry’). This new approach provides transparent feedback during self-regulation training, and it has the potential to enable more specific training effects for future therapeutic applications such as clinical interventions in mood disorders.

This article is part of the theme issue ‘Neurofeedback: new territories and neurocognitive mechanisms of endogenous neuromodulation’.

## 1. Introduction

Functional magnetic resonance imaging (fMRI) neurofeedback (NF) enables participants to learn to self-regulate activity in brain areas and networks using information extracted in real-time from those regions. This exciting possibility has stimulated an increasing number of fundamental and clinical NF applications although only limited information, such as the strength of activity or a single classifier confidence value has been provided as feedback in most studies. In this future perspective article, we present a novel approach, called semantic fMRI neurofeedback (sNF), that is based on representational similarity analysis (RSA) [1] of evoked brain activity patterns extracted from brain areas coding semantic information. We argue that providing *semantic information* to participants about the similarity of their current mental state (CMS) to other related mental states, instead of merely providing the strength of activation, enables more specific and effective self-regulation training. As a specific application, we discuss sNF in the context of clinical neurofeedback interventions for mood disorders, especially depression [2–4]. We will describe how sNF differs from conventional fMRI neurofeedback, review first results from non-clinical sNF studies using object imagery and discuss how we envision the application of sNF for the treatment of mood disorders in the future.

In conventional activation-based fMRI NF [5], a participant receives ongoing information about the average amount of activity relative to a baseline level in a selected brain region or network, typically in a thermometer-like display. Based on the received feedback, a participant may learn to influence the feedback activity strength value by learning to engage in a specific mental task (e.g. recalling and engaging in emotional autobiographical memories) that activates the selected region (e.g. voxels in emotion-processing areas). During self-regulation training, the participant adjusts the mental task (e.g. by engaging more vividly in an emotional autobiographical memory or by recalling another emotional memory) and thereby usually improves the ability over time to activate the selected brain region. Learning to modulate the activity in this way can lead to beneficial effects as assessed using relevant outcome measures [6]. Conventional fMRI NF has e.g. achieved a substantial reduction of symptoms in patients with depression as assessed with standard clinical instruments [2–4].

One main open question is that of specificity. In our previous study [4], neurofeedback with an emotional localizer was not superior to a control intervention targeting the parahippocampal place area (which is modulated by imagery of houses and scenes). RSA and semantic neurofeedback have the potential to identify and target brain patterns of specific emotions and thereby more directly target individual deficits in emotional expression and experience. At a more general level, patients in previous emotion–regulation neurofeedback studies were instructed to engage in positive feelings, but the employed conventional (univariate) neurofeedback was ‘blind’ with respect to the emotional content that participants engaged in. For instance, the feedback mean activity level could be driven by emotional content with negative valence while the experimenter would assume that the increased activity in emotion-related areas is caused by positive emotional mental states. Such a possibility could only be confirmed (or ruled out) during post-scan debriefing. This blindness to the emotional contents is related to the neuroimaging finding that different emotional mental states (e.g. ‘happy’ versus ‘sad’) are associated with strongly overlapping activity in emotion-related brain areas and are, thus, difficult to disentangle using univariate analysis approaches [7–9]. To discriminate between discrete emotions or characterise emotional properties along one or more affective dimensions, such as valence and arousal, multivariate pattern-based analysis methods seem to be necessary [8–12]. However, to be applicable for real-time semantic neurofeedback training, studies are needed to investigate whether differential multivariate patterns evoked by different emotional mental states can be obtained within short temporal windows of a few seconds and robustly visualized in a semantic map (see §2). One way to enhance the chance to discriminate multivariate patterns of different emotions in a short time window is to employ fMRI at ultra-high field strengths (7 Tesla and higher), making it possible to sample robust and fine-grained discriminative activity patterns at higher spatial resolution and/or higher signal-to-noise ratios [13–16]. In a series of proof-of-concept studies using mental imagery of objects from different categories (cat, dog, chair, hammer), our research team could demonstrate that 3 Tesla and especially 7 Tesla fMRI provide enough signal-to-noise and spatial resolution to extract fine-grained semantic information from higher-level visual cortex during real-time fMRI [17,18]. Whether this also holds for semantic neurofeedback of emotional mental states has, however, not yet been demonstrated and is currently being evaluated.

## 2. Methods

**Figure 1.**
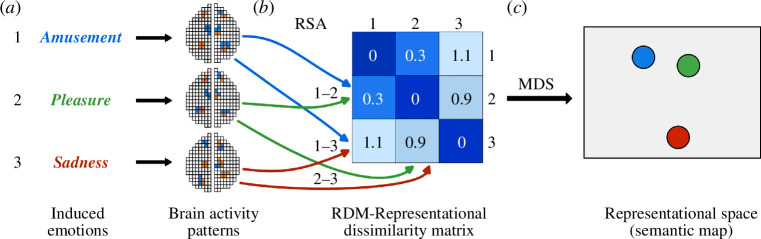
Illustration of representational (dis)similarity analysis and semantic map creation with *n* = 3 conditions of induced emotions. (*a*) Presented stimuli (e.g. pictures) or engaged emotional autobiographical memories evoke *n* = 3 distributed activity patterns. For voxels in a selected region of interest (ROI; e.g. in the ventral prefrontal cortex), the *n* patterns are compared pairwise, usually by calculating a linear correlation value. (*b*) Similarity (e.g. correlation) values are converted into dissimilarity values (*d*), e.g. *d* = 1 – correlation, ranging from perfect correlation (*d* = 0) to no correlation (*d* = 1) to perfect anticorrelation (*d* = 2); pairwise dissimilarity values are placed in respective cells of the representational dissimilarity matrix (RDM). Besides numbers, the values in the RDM cells are often visualized using a colour range (here: dark blue, low dissimilarity; light blue, high dissimilarity). (*c*) Using multi-dimensional scaling (MDS), the high-dimensional similarity information encoded in the RDM is projected onto a two-dimensional representational space or semantic map.

Semantic neurofeedback uses RSA [1] to differentiate and relate content from overlapping fMRI brain activation patterns extracted from brain areas coding semantic information. Semantic neurofeedback requires at least one preparatory (localizer) run prior to subsequent NF runs to establish a representational space (semantic map) of induced mental states, e.g. ‘neutral’, ‘happy’, ‘sad’, ‘angry’, etc. in case of emotional mental states. During sNF, the calculated representational space will be shown to the subject together with a point indicating the position of the CMS. The representational space is created using RSA, i.e. by calculating the pairwise (dis)similarities of evoked response patterns extracted from voxels in relevant brain regions (figure 1*a*,*b*). Content-specific activity patterns (e.g. related to different emotions) can be induced in various ways, e.g. by using standardized picture sets or by asking participants to engage in mental tasks such as emotional autobiographical memories [16,19,20]. The dissimilarity information is then visualized using multi-dimensional scaling (MDS [21]), placing each mental state at a location in a two-dimensional space that maximally satisfies the pairwise dissimilarities of all conditions (figure 1*c*).

**Figure 2.**
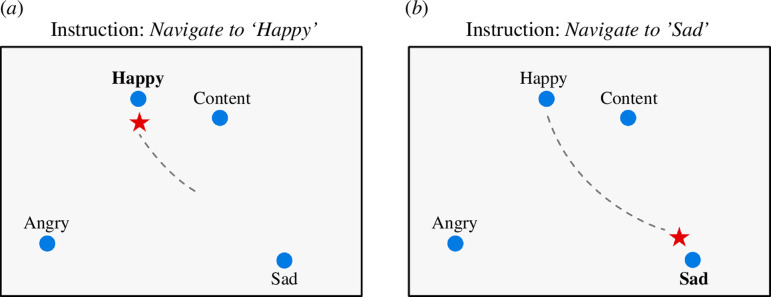
Schematic illustration of the visualization of navigated emotional mental states as provided to the participant. The (individual) semantic map contains the four emotions ‘happy’, ‘content’, ‘angry’ and ‘sad’. After receiving the instruction ‘navigate to “happy”’, the participant engages in a happy emotion and the CMS point (red star) moves from a starting (‘neutral’) point towards the location of the ‘happy’ emotion in the semantic map (*a*). After subsequently receiving the instruction ‘navigate to “sad”’, the participant engages in a sad emotional state and the CMS point moves to the location of the ‘sad’ emotion in the semantic map (*b*). Note that in case the participant engages in a negative (e.g. sad) emotion while being instructed to navigate to a positive (e.g. happy) emotion, this could be detected online and acted upon.

During semantic neurofeedback (figure 2), the CMS of the participant is placed in the two-dimensional semantic map as a point (red star in figure 2) indicating the semantic distance to landmark points representing the emotional mental states measured in the preparatory runs. The participant is then instructed to engage in specific mental tasks (e.g. evocation of feelings related to different emotions), and the changing mental state will be reflected in movements of the CMS point towards a landmark point on the map that corresponds to a desired mental state (e.g. ‘happy’).

### (a) Feedback of pattern similarity and pattern intensity

The feedback signal in most conventional fMRI-NF studies is a univariate summary signal that reflects the mean activity of voxels in a specified region of interest (ROI) [22]. The univariate summary signal is usually provided to the participant in a thermometer-like display, which has been proven easy to comprehend by participants [23]. While providing useful information to participants, using the mean activity for feedback has the disadvantage that it merely reflects the intensity of activity (e.g. emotional engagement), rather than the content of the mental state (e.g. ‘happy’ versus ‘sad’ emotion). Thus, univariate activation-based NF only provides information about the up-regulation or down-regulation of a region or network but it does not reveal what specific emotional representation the participant regulates. The strategies employed by participants could, thus, be non-specific. For example, successful self-regulation of a mean activity signal from emotion-related regions could be related to undesired emotions or to altering arousal levels rather than specific desired (e.g. positive) emotional states. RSA-based semantic neurofeedback, on the other hand, encodes multivariate voxel patterns providing information about the similarity of the CMS with respect to voxel patterns of emotional mental states measured during localizer runs.

**Figure 3.**
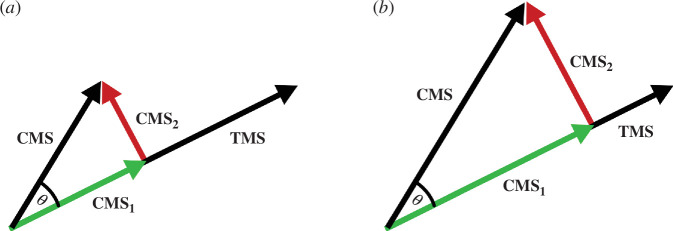
(*a*) Calculation of the pattern intensity of the CMS vector with respect to a selected TMS vector. Projecting vector CMS on TMS provides vector CMS_1_. The length of vector CMS_1_ relative to the length of target vector TMS equals the normalized intensity of the CMS relative to the TMS and is referred to as the ‘target-related pattern strength value’. (*b*) Same correlation similarity (same angle *θ*) as in (*a*) but stronger intensity of the CMS vector resulting in a stronger target-related pattern strength value **CMS**_**1**_.

To assess the similarity between pairs of activity patterns from the same ROI, the correlation distance (i.e. 1 − Pearson’s correlation) is often used in the context of RSA. While there are other similarity/distance measures such as Euclidean (order 2 Minkowski) or Mahalanobis metrics [24], the correlation distance is particularly well suited for real-time fMRI applications because of its efficient calculation and intuitive interpretation. This metric exploits the information conveyed by the angular distance between patterns/vectors, disregarding the amplitude of the activation. Indeed, the correlation metric normalizes for both the mean and standard deviation of the pattern activity distribution. It might, however, be beneficial to feedback on both, pattern similarity and some ‘intensity’ information for self-regulation training. If in an idealized scenario, a participant recreates the pattern of a desired emotion (e.g. ‘happy’) and then continues to engage more strongly in that emotion, one would observe a scaled response of voxel activities (ignoring noise). Since pattern similarity measured using Pearson’s correlation would not change because of the normalization properties of the metric, such further engagement in the emotional state would not be visible in the feedback display, i.e. the distances the CMS points to the anchor states would not change. For more comprehensive feedback reflecting both similarity as well as intensity, it might be beneficial to include information about the activation strength of a pattern, e.g. by abstracting an activity pattern into a numeric vector and defining the pattern strength as the vector length. More specifically, we propose to quantify the activation strength of the CMS pattern with respect to the activation strength of the (closest) target mental state (TMS) pattern (as measured in localizer runs) by projecting the CMS vector on the TMS vector (figure 3). The length of the projected vector relative to the length of the target vector provides the target-related pattern intensity value. The calculated intensity value can be used to enrich the visualized feedback provided to the participant, e.g. by reflecting the intensity value by the size or colour of the CMS point. The idea to include and visualize information about both pattern similarity and pattern intensity during neurofeedback will be discussed in §4.

Additionally, other dissimilarity metrics, such as Euclidean or Mahalanobis distance, might still be considered as they inherently incorporate the magnitude information. Furthermore, previous analytical studies have shown that these metrics are more robust to spatial noise than correlation distance and could better capture the underlying dissimilarity structure of brain patterns [24,25]. However, whether and to what extent the highlighted properties might be beneficial for semantic neurofeedback still needs to be investigated. Future empirical studies will be needed to assess which metric provides the best information for successful self-regulation training.

### (b) Relationship of semantic neurofeedback to classifier-based neurofeedback

Like semantic neurofeedback, classifier-based NF [26] uses multi-voxel pattern analysis (MVPA) to detect differences between neural correlates of different mental states with higher sensitivity than conventional univariate statistical analysis. In one or more runs during a training phase, weights are estimated for each voxel to build optimal decision boundaries between classes. Typically, linear classifiers like support vector machine (SVM) are employed to analyse real-time fMRI data (e.g. [12,26]). During neurofeedback runs, after the classifier training, activation patterns evoked by participants are classified online. The prediction output produced by the classifier (e.g. a value < 0 for class A, a value > 0 for class B) can be converted into a feedback value for the participant indicating the likelihood that the current distributed activity pattern belongs to a given class. While pattern classifier NF studies have extended the type of data extraction and analysis over univariate analyses, most NF studies have used binary classifiers with one-dimensional feedback information indicating whether the current activity pattern generated by the participant belongs more to class A or more to class B [27–29].

Decoded neurofeedback (DecNef [30]) is a variant of classifier-based NF focusing on unconscious reinforcement learning, usually with monetary reward [31–33]. More specifically, DecNef is based on an implicit approach that does not require conscious knowledge of participants about what they are supposed to do or learn [31–33]. Explicit neurofeedback training is replaced with associative learning by pairing multi-voxel patterns with online reward (or online punishment or another stimulus) and participants learn to generate patterns maximizing the reward. To relate reward to a specific brain process, it is, however, necessary to train a multivariate predictive model (decoder) in a training session (called the decoder construction session) in the same way as described for classifier-based neurofeedback.

As both classifier NF and DecNef leverage the analysis of multi-voxel patterns within target ROIs, they are related to semantic neurofeedback in that they also aim at increasing content specificity by exploiting the local spatial distribution of neural activities and by reducing the high spatial dimensionality of fMRI data sets. The added value of semantic NF consists in the use of RSA and MDS to explicitly attempt to convert the spatial patterns into a *continuous representational space*. Indeed, a continuous semantic space for emotion-related feedback avoids setting boundaries among different emotional classes [8,34], which has proven to be challenging [35]. Note that it might be useful to combine classifiers and semantic NF: multi-voxel pattern classifiers could serve as a pre-processing (voxel selection) step for subsequent RSA detecting those features from the selected ROI in the localizer run(s) with the highest discriminative weight values.

## 3. Previous semantic neurofeedback studies using object imagery

As mentioned earlier, two proof-of-concept studies have been performed to evaluate the semantic neurofeedback approach. These first studies did not use representations of emotions but focussed on the semantic representation of imagined visual objects based on activation patterns extracted from the ventral visual cortex [17,18]. The experimental design included four functional runs (pattern generation runs) prior to the NF runs, in which participants were requested to generate a semantic representation of four concrete objects via mental imagery. Local fMRI patterns of activity for each object were extracted from the left ventral temporal cortex and RSA was applied to analyse the representational structure of the four neural patterns. Namely, pairwise pattern dissimilarities were evaluated as correlation distances (1 − Pearson’s correlation) and resulted in a 4 × 4 RDM. The representational structure encoded in the RDM was then translated into a two-dimensional representation using an MDS procedure [36]. In the two-dimensional representational space, the four base patterns were represented as labelled dots whose mutual distances reflected the correlation distance computed among the respective neural patterns. During the subsequent NF runs, participants were asked to replicate the neural representation of one target object. Real-time RSA and MDS were employed to generate visual feedback, where the CMS of the participant was represented as a moving point on the semantic map, whose position changed according to the correlation distances computed between the online brain pattern and the base (object-specific) patterns. The position of the current pattern was estimated using the landmark MDS approach proposed in de Silva & Tenenbaum [37]. Thanks to this projection procedure, the participants were informed about the ‘position’ of their CMS with respect to the base patterns and could adjust their imagery strategy to navigate as close as possible to the target pattern.

Our previous studies demonstrated the feasibility of the proposed semantic neurofeedback approach to guide the modulation of a target pattern using both 3 T and 7 T MRI. Furthermore, at the group level, participants improved their ability to replicate the TMS within the NF runs and between the first and the second NF runs, corroborating the more general idea that providing semantically driven feedback enables the self-regulation of mental states in a multi-dimensional space.

## 4. Towards clinical applications

While semantic neurofeedback has been successfully applied to navigate a semantic map of object categories using patterns from the ventral visual cortex (see §3), it has not yet been applied to emotional contents. Future research needs to show that disentanglement of several evoked overlapping emotion patterns and calculation of a robust semantic map is feasible online with this method, at least at an individual level. Furthermore, it needs to be assessed whether the measured current emotional state can be placed as a point inside an individual emotional semantic map in a robust way to support transparent and efficient self-regulation training. In the following sections, we discuss these and other relevant questions that are subject to current and future research with the goal of enabling semantic neurofeedback training for clinical interventions of mood disorders.

### (a) Induction of emotions

During semantic neurofeedback participants mentally evoke emotions. However, it needs to be decided how to evoke emotions during the preparatory runs to build individualized emotional representational spaces that are then used during sNF. Temporary mood states can be induced through a range of methods, including the presentation of visual stimuli, musical excerpts, imagery or autobiographical recall [38,39]. In the experimental setting of the fMRI laboratory, visual stimulation has probably been used most widely (both static images and movie clips). The effectiveness of different emotion induction methods has been largely debated. Different categories of affect inductions could yield effects of various intensities depending on the target emotion [39,40]. A recent study [41] found that different induction methods elicited different profiles of neural activity, thus suggesting that the target network should also be considered in the choice of the emotion induction approach. Previous studies [42] showed that personalization of the stimuli using, e.g. individual ratings, can be relevant to the induction of emotion-specific brain activation patterns. Based on the available literature and recently conducted pilot studies inducing up to nine positive emotions [16], we aim to use autobiographical memories for emotion induction in future applications of sNF for emotion regulation. Indeed, autobiographical memory recall has been proven to trigger intense emotional reactions and is a flexible tool for emotion-regulation interventions as it enables emphasis on both positive and negative details of past personal experiences. Furthermore, previous studies have shown that the recall of specific positive autobiographical memories facilitates mood regulation, reducing negative emotions and enhancing positive moods [43,44].

**Figure 4.**
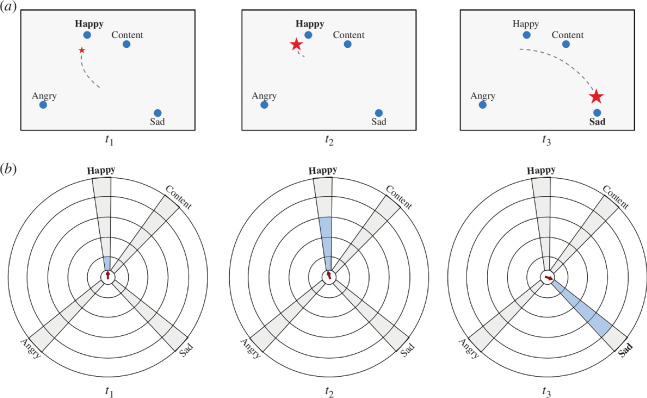
Different visualization approaches combining feedback of pattern similarity and pattern strength illustrated using three time points (*t*_1_, *t*_2_, *t*_3_). The TMS is indicated by bold text of the respective emotion label. (*a*) In this variant, a semantic map is shown with distances calculated using MDS as in pilot (object imagery) studies; pattern strength is, however, integrated by drawing the CMS point (here: red star) in different sizes. In *t*_1_ and *t*_2_, the similarity of the CMS pattern to the TMS (‘happy’) does not differ much but the pattern strength has substantially increased in time point *t*_2_ as compared with *t*_1_ as indicated by the size of the red star. The star is even larger in time point *t*_3_ after the participant navigated to TMS ‘sad’. (*b*) In this proposed variant, a circular semantic map highlights pattern strength using a thermometer-like display with emotions placed in a circular arrangement. The three different time points correspond to the same scenarios as depicted in (*a*). The red arrow in the middle reflects pattern similarity (for details see text). In scenario *t*_1_, the CMS pattern is close to the ‘happy’ mental state pattern, but pattern strength is weak as indicated by the low filling of the ‘happy’ thermometer (blue colour). In scenario *t*_2_, pattern similarity is similar as in *t*_1_ but pattern strength has increased substantially. In *t*_3_, pattern intensity with respect to target pattern ‘sad’ is stronger than in scenario *t*_2_.

Classical functional neuroimaging studies of emotions have often used representations of discrete ‘basic emotions’, e.g. in a prototypical set of facial [45] and bodily [46] expressions. Using this type of emotion mapping, one would aim to obtain brain activation patterns related to individual emotions, which might then order along a bidimensional coordinate system, for example along dimensions of valence and arousal in a ‘circumplex’ representation [47,48]. Such a dimensional representation matches well with the use of image databases that are based on an explicit valence/arousal dimensional model of emotions such as the International Affective Picture System (IAPS [49], or Open Affective Standardized Image Set (OASIS) [50]). Using (individually rated) images from such databases would ideally yield brain activation patterns that can be used as anchors for the different states in a multi-dimensional emotion system (for example, ‘positive valence, high arousal’, see also figure 4*b*). The creation of a semantic map of emotions using RSA and MDS as used in sNF can be seen as an individualized data-driven approach integrating discrete and dimensional theories of emotion [51,52].

### (b) Visualization of semantic emotional feedback

After the creation of an individualized semantic map of emotions using localizer runs, the CMS is dynamically projected onto the two-dimensional semantic map during sNF runs. As discussed earlier, there are different possibilities to visualize the similarity of the CMS to the base (anchor) emotional mental states. In our previous sNF studies targeting object representations, the semantic map produced by MDS was used, and the CMS was shown as a moving point within that map based only on pattern similarity calculated using Pearson correlation (figure 2). As discussed above (§2a), a useful addition to a visualization based on normalized pattern similarity would be the integration of the pattern intensity of the CMS vector. A simple modification of the semantic map would be to reflect pattern intensity by adjusting the size or colour of the moving point (figure 4*a*). Note that the size (or colour) of the CMS point in this modification is determined by calculating the projection of the CMS vector on the target vector as described above (figure 3). The target emotion vector chosen at any given feedback moment is the one with the highest similarity (correlation) to the CMS vector.

While incorporating information about pattern strength, the visualization proposed in figure 4*a* is reflecting mainly pattern similarity. Alternative visualizations could focus more on pattern strength. Figure 4*b* shows a proposed design presenting several thermometers, each representing a different pre-mapped emotion, in a circular arrangement. Here pattern strength is visualized by the degree of filling within a thermometer. The placement of thermometers around the circle could approximate the measured dissimilarities between base mental state vectors or follow a generic semantic arrangement such as the two-dimensional valence-arousal model [47]. In figure 4*b*, emotions have been placed roughly along these two dimensions with positive valence on top, negative valence at the bottom, low arousal on the right and high arousal on the left side. To avoid information overload, the filling of only one thermometer is shown at any moment in time. This ‘target’ thermometer is determined as the one with the highest pattern similarity of its corresponding base emotional state vector with the CMS vector. While the filling of the selected thermometer reflects pattern strength, pattern similarity could be optionally visualized by an arrow in the centre of the circular display (figure 4*b*). The arrow would point between the target thermometer and the neighbour (left or right) with the largest correlation value. To obtain the exact point on the inner circle at which the arrow points, a distance measure is first calculated as ‘*dist* = 1 − (*target-similarity*/(*target-similarity + max-neighbour-similarity*)’. This measure provides the pattern similarity of the CMS vector and the mental state vector of the most similar neighbour. The calculated *dist* value is then multiplied by the angle between the target and the selected neighbour thermometer, providing the point highlighted by the displayed arrow.

The two presented (or similar) visualization approaches need to be assessed and compared in future semantic neurofeedback pilot studies. Using the semantic map variant enhanced with the size (or colour) of the CMS point (figure 4*a*) has the advantage that it is very similar to the representation successfully used in our previous (object imagery) pilot studies [17,18]. The variant with multiple thermometers in a circular arrangement (figure 4*b*) highlights pattern strength more explicitly but it is visually more complex and would only be chosen if it would lead to a more successful emotion-regulation training than the enhanced MDS-based semantic map variant.

## 5. Discussion and conclusions

In this future perspective, we presented semantic neurofeedback as a new variant of self-regulation learning of emotions using real-time fMRI that introduces the idea of using RSA and MDS for neurofeedback with the goal of providing transparent information about the CMS with respect to relevant ‘anchor’ emotional mental states. This new approach enables participants to navigate in a continuous representational space as has been demonstrated in the case of visual object imagery using data from a high-level ventro-temporal visual cortex known to code semantic information of objects [17,18]. We have described the methods developed and tested for these pilot studies that pave the way for clinical applications of sNF using emotional semantic content. We envision the application of sNF primarily for mood disorders, especially depression treatment, because of previous encouraging results when using conventional neurofeedback [2–4] and because the self-regulation of emotions is an important aspect of therapeutic interventions more generally. We presented different ways of how one could visualize a continuous individualized representational space of emotions based on pattern similarity in relevant brain regions that optionally may also include information on the strength of the ongoing modulated mental state pattern. This form of neurofeedback also promises to better track a patient’s progress during self-regulation training as assessed by improvements over time in navigating the emotional representational space. This information and the underlying modulation of multivariate patterns will provide rich fine-grained information on self-regulation learning that can be related to changes in pre- and post-clinical assessment scores. In parallel, arousal and stress levels should be assessed via the simultaneous collection of physiological signals, which can also be used as potential covariates in the analysis of clinical and neuroimaging data. Moreover, while the accurate mapping of the emotional space remains challenging, the data-driven construction of an individual (emotion) similarity space for participants implied by the semantic neurofeedback approach could help to overcome some of the limitations and simplifications of previous approaches. Although it is thus likely that semantic neurofeedback can target emotional states more specifically than conventional neurofeedback, the induced emotional mental states during the preparatory runs used to create the semantic map would still need to be checked against participants’ self-report of experienced emotions, at least for clinical applications, to ensure that patients indeed engage in the clinically desired emotional processes.

While two physical dimensions are the strict minimum required to produce visual semantic maps of emotional patterns, more than two dimensions, e.g. a third spatial coordinate in the visual space introducing a depth to the representational space and/or a frequency or amplitude modulation of an auditory signal to involve auditory perception, could be exploited to produce enhanced (multi-)sensory semantic maps. Indeed, using more than two dimensions would also allow for reducing the distortion on the distances when projecting a higher dimensional space to a sub-space implied by any MDS-like approach applied to RDMs (see [17,53] for a critical discussion on this more technical aspect). Additionally, a correlation analysis between the neurofeedback performances and the goodness of the low-dimensional embedding of the semantic information, as assessed by stress values [54], would be particularly useful to further investigate the efficacy of the low-dimensional feedback. More generally, the flexibility of the semantic neurofeedback method with respect to the experimental paradigm needs to be further investigated. This includes, e.g. the use of intermittent or continuous feedback, which can both be used in combination with semantic neurofeedback.

Establishing the novel real-time fMRI semantic neurofeedback approach to emotion regulation will likely provide new insights into the distributed representation of emotions, especially in the prefrontal cortex. Semantic NF studies could, thus, serve as a research tool to gain new insights into the similarity structure of internally generated mental representations and thereby complement results from externally stimulated paradigms.

Semantic neurofeedback will likely also provide unprecedented transparency of the ongoing self-regulation process for both the participant and the experimenter. While this perspective focuses on depression and mood disorders, the applicability of semantic neurofeedback might be broader and could be evaluated in the context of other psychiatric diseases. More generally, semantic neurofeedback might develop into a (clinical) imagery training technique supporting patients (as well as healthy individuals) to develop and exercise specific ‘healthy’ mental states. Such a training approach would be highly relevant since proficient imagery performance plays an increasingly important role in the treatment of diseases such as PTSD and depression (e.g. [55]).

## Data Availability

This article has no additional data.
